# HPV knowledge and impact of genital warts on self esteem and sexual life in Colombian patients

**DOI:** 10.1186/1471-2458-13-272

**Published:** 2013-03-25

**Authors:** Marion Piñeros, Gustavo Hernández-Suárez, Liliana Orjuela, Juan Carlos Vargas, Gonzalo Pérez

**Affiliations:** 1 Fundación para la Investigación y el Desarrollo, Bogotá, DC, Colombia; 2 Profamilia, Bogotá, DC, Colombia; 3 Merck Sharp & Dohme, Whitehouse Station, New Jersey, USA

**Keywords:** Genital warts, HPV, Psychosexual impact, Patients, Colombia

## Abstract

**Background:**

Information on HPV knowledge in patients with genital warts is scarse as is the information on factors related to the impact on self-esteem and sex life among them.

**Methods:**

We conducted a cross-sectional study in adult patients with a clinical diagnosis of genital warts (GW) attending a major private out-patient clinic in Bogotá, Colombia. Patients underwent biopsy for pathological diagnosis, HPV-DNA testing and completed a questionnaire assessing HPV knowledge, and the consequences of GW on self-esteem and sexual life. Differences in proportions were assessed with a chi^2^ test.

**Results:**

106 men and 155 women had pathologic confirmation of GW. 51% of subjects had heard of HPV before consultation coming mainly from the media (82%). Less than half of the participants knew that HPV could be transmitted through non-penetrant sexual intercourse and only two thirds acknowledged HPV vaccine as a preventive measure against HPV infection. Impact on self-esteem was higher among women than men (90.3% vs 60.4%, [p < 0.01]). In men, factors related to a higher impact on sexual life were HPV awareness and age; in women they were higher education and anatomic location; external GW had a higher impact on sexual life in women (83% vs. 66%; [p = 0.05]).

**Conclusions:**

We found a low awareness of HPV and low knowledge on the vaccine as a preventive measure for associated diseases even in patients suffering from genital warts, highlighting the need for communication and education on HPV. Greater impact on self-esteem in women might reflect higher health consciousness among Latin American women.

## Background

Genital warts (GW) are caused by infection with certain types of human papillomavirus virus (HPV) and are one of the most prevalent sexually transmitted infections in the world [1,2]. The disease affects mainly adolescents and young adults, who are more sexually active and therefore susceptible to primary HPV infection after the onset of sexual activity [1].

Although information on the prevalence of genital warts is scarce, it is estimated to be approximately 1% among the sexually active populations in some high-income countries. Data also suggest that the cumulative lifetime risk of GW can reach around 10% [1,3,4]. In addition, several epidemiological studies show that the prevalence of genital warts seems to be increasing [4].

The recent development of prophylactic HPV vaccines that provide protection from HPV types 6 and 11 (types implicated in more than 90% of genital warts) [5] is a potential benefit for the reduction in the burden of GW in populations where HPV vaccination with the quadrivalent vaccine has been introduced. This benefit has been demonstrated in the Australian population since the start of HPV vaccination [6]. The assessment the benefit of HPV vaccination with respect to GW in other populations will likely follow based on the prevalence of the disease, the related costs and a better understanding of the knowledge regarding HPV and its health consequences.

In the Latin American or Hispanic population, despite the high burden of cervical cancer in the region [7], little is known concerning either the burden of GW or the general population’s knowledge of HPV and related diseases. A systematic review of HPV knowledge that included 39 studies showed that only two of them had been carried out in Latin America [8]. More recently, other studies have dealt with this issue in Colombia [9,10].

Importantly, the proportion of persons that have heard about HPV varies between studies (13% to 95%), but most report that, in general, the knowledge of the virus remains lacking [8] although after the introduction of the vaccine it has improved substantially [11,12]. In Colombia, HPV vaccine was introduced in 2012 in a school-based program; a nation-wide survey performed in 2010 revealed that among women between the ages of 18 and 69, 44% were aware of HPV and 25% had heard about the HPV vaccine [13]. Awareness of the association between HPV and GW was explored in a Brazilian study showing that only 5% of interviewed persons had knowledge of the virus’ association to GW [14].

Data indicate that patients with GW suffer anxiety, experience shame and carry the stigma of having a venereal disease. All of these disease sequelae affect sexual and love lives as well as health-related quality of life [15-18], though there are little empirical data on how psychological distress related to GW impacts quality of life [19,20].

The aim of this study was to examine awareness and knowledge of both the HPV virus and the HPV vaccine, as well as explore how genital warts affects self esteem and sexual life in patients with genital warts attending an outpatient clinic in Bogotá, Colombia.

## Methods

### Study design and participants

The present report was part of a broader study entitled “Prevalence of HPV genotypes in genital warts of a Colombian population” that was approved by the Ethical Review Board of Profamilia ^a^ in Bogotá, Colombia.

Between December 2009 and August 2010 we invited male and female patients, aged 18 to 45 years, who lived in Bogotá at the beginning of the study and who attended the clinic with lesions suspected of GW. Patients with clinical diagnosis of GW were invited by the gynecologist or urologist in the first consultation to participate in the study and consented their participation; all patients underwent a biopsy for the establishment of the associated HPV genotypes and afterwards a questionnaire was applied by a trained interviewer. Patients not willing to participate, having received the HPV vaccine or with a known diagnosis of an immune suppressive disease were excluded from the study. The questionnaire included sociodemographical aspects, awareness and knowledge of HPV and HPV vaccine, sources of HPV-related information, initiation of sex life and previous medical history of STDs. Clinical features of the lesions diagnosed were also registered.

Affiliation to the Colombian Health System was defined according to the law in three broad regimes: ‘contributive’, corresponding to workers or their families, where a proportion of the salary goes to the payment of the insurance; ‘subsidized’ corresponding to persons and their families of lower economic conditions where the government subsidizes their basic health care and ‘non-affiliated’ for those who at the moment of the clinical diagnosis were not covered by the health system.

Awareness of HPV and HPV vaccine was assessed through the question whether patients had heard about HPV (or HPV vaccine) before coming to the clinic. Knowledge was assessed through a series of “false or negative” statements on HPV transmission, on HPV related diseases (cancer and genital warts) and on preventive measures (vaccination, use of condom, number of sexual partners). To evaluate the effect of GW on self-esteem we included the following question used in a qualitative study [20]. “Have GW affected your self-esteem?” with a ‘yes/no’ option answer. In addition to assess the impact of GW on sexual life we used a visual analogous scale where “0” reflected “no concern”, ratings from 1 to 3 “mild” impact, from 4 to 7 “moderate” impact and from 8 to 10 “severe” impact. The questions and level of concern were selected considering previous reports on impact of genital warts [2,20].

Physicians involved in the study were given a short training on general conceptual and operative aspects of the study. A pilot study with 20 patients was carried out for adjustment of questionnaires and general procedures.

### Statistical analyses

Frequencies were presented as absolute values and percentages. Differences in proportions, were assessed with a chi^2^ test. A multinomial lineal model was used to evaluate what variables were associated with self-esteem, calculating prevalence ratios and 95% confidence intervals. A multivariate model was used to check whether there was confusion between variables related to awareness of HPV and HPV vaccine. All statistical procedures were performed using STATA® software.

## Results

The study included 342 patients (139 men and 204 women) with genital lesions suspected to be GW, with a participation rate of 99% (Only three patients refused to participate). From these, 106 (77%) men and 155 (76%) women had pathologic confirmation of GW and all responded the survey and were included into the analysis

45% of the study population was between 25 and 34 years old and 40% was 18 to 24 years old. The majority (71,7%) pertained to the contributive health regime. Half of the men and women had heard about HPV before consultation (Table 1). There were no significant differences in HPV awareness according to gender, age and number of life-time sexual partners. On the contrary, awareness of HPV was higher among patients with higher education, married persons and in those affiliated to the contributive regime.

**Table 1:** Awareness of HPV and HPV vaccine among study population

	**HPV awareness**				**HPV vaccine awareness**		
**Characteristics**	**Total**	**Yes**	**%**	**p Value**	**Yes**	**%**	**p Value**
**Sex**							
Male	106	53	50.0		28	26.4	
Females	155	76	49.0	0.87	75	48.4	<0.01
**Age**							
18-24	105	48	45.7		40	48.1	
25-34	117	62	53.0		48	41.0	
35-44	39	19	48.7	0.55	15	38.5	0.70
**Civil Status**							
Single	174	87	50.0		68	39.1	
Married	53	31	58.5		24	45.3	
Widow/Divorced	10	5	50.0		6	50.0	
Cohabitation	24	6	25.0	0.10	5	20.8	0.20
**Education Level**							
Primary	17	3	17.6		0	0.0	
Secondary, High-school	154	66	42.9		51	33.1	
University	61	40	65.6		36	59.0	
Postgraduate	29	20	69.0	<0.01	16	55.2	<0.01
**Social security**							
HMO-workers	187	100	53.5		84	44.9	
Subsidary	11	3	27.3		3	27.3	
No Affiliation	63	26	41.3	0.08	16	25.4	0.01
**Genital Warts**							
Incident	245	119	48.6		95	38.8	
Recurrent	16	10	62.5	0.27	8	50.0	0.37
**Life-time sexual partners**							
1 to 4	125	59	47.2		59	47.2	
5 to 9	77	38	49.4		27	35.1	
10 or more	59	32	54.2	0.36	17	28.8	0,03
**Information Source**							
Media (newspaper, TV, radio	87	72	82.2		52	59.8	
Friends, family	29	21	72.4		13	44.8	
Health personnel	28	15	53.6		12	42.9	
School, university	18	14	77.8		9	50.0	
Never heard	92	3	3.3	n/a	14	15.2	n/a

n/a categories not mutually excluded.

Only 25% of men had heard about the HPV vaccine, though this measure was also significantly higher among patients with higher education, affiliated to the contributive regime, and among those with 1 to 4 life-time sexual partners. The main source of information in both men and women was the media (82%) (Table 1). Awareness of HPV as well as of the HPV vaccine was higher among patients with recurrent warts, although the difference was not statistically significant. The multivariate model yielded no confounding in variables related to awareness (results not shown).

Among those who had heard about HPV, virtually all knew that HPV was a sexually transmitted disease, although less than 50% of men and women knew that the infection can also be passed through non-penetrant sexual intercourse and almost 60% responded erroneously that HPV was also transmitted trough contact with infected objects (Table 2). The majority of patients knew that the use of condom would reduce the probability of infection with HPV (slightly more women than men 81% *versus* 91% (*p* value 0.02)(Table 2) Almost all patients (98%) knew that the HPV infection causes genital warts and 82% knew that it causes cervical cancer. Only 60% of the patients identified the vaccine as a way to reduce infection (Table 2).

**Table 2:** Knowledge of HPV among male and female patients with genital warts

	**%**	
**Knowledge**	**Men**	**Women**
**HPV transmission**		
Passed through penetrant intercourse (true)	98.1	98.6
Passed trough non-penetrant sexual intercourse (true)	42.5	45.2
Passed trough contact with infected objects (false)	59.4	59.4
**HPV related diseases**		
Genital warts (true)	98.1	98.7
Cervical cancer (true)	77.4	98.7
**Prevention/reduction of HPV infections**		
One sexual partner (true)	79.3	71
Use of condom (true)	80.8	90.7
Vaccine (true)	58.8	65.8
Personal hygiene (false)	53.8	54.2

n/a categories not mutually excluded.

In response to the question “Have GW affected your self-esteem?”, 90% of women and 62% of men responded affirmatively (p < 0.01). When exploring the variables that might be associated with this outcome by sex, none of them showed a statistically significant association (Table 3).

**Table 3:** Multivariate prevalence ratios (PR) of factors associated with impact on self-esteem in men and women

**Factors**	**Women**		**Men**	
	**PR**	**CI95%**	**PR**	**CI95%**
Awareness of HPV	1.06	[0.75-1.51]	1.59	[0.92-2.73]
Age	1	[0.97-1.03]	1.03	[0.99-1.07]
Education level	0.99	[0.71-1.36]	0.87	[0.61-1.25]
Life- time sexual partners	0.98	[0.70-1.39]	0.82	[0.56-1.20]
Anatomic location*				
**Women**				
Vulva Introitus	0.83	[0.58-1.19]		
Perineum	1.04	[0.48-2.27]		
**Men**				
Glans/sulcus			1.09	[0.56-2.10]
Foreskin			0.68	[0.35-1.32]
Perineum			0.72	[0.25-2.09]

*Labia and shaft were the reference category (as the most frequent) in each sex.

Women with GW were more likely to report a severe impact on sex life than men (77% vs 46%, [p < 0.001]). Factors associated with this outcome were not the same by sex. In men, both age and knowledge of HPV were related to a severe impact on sex life. Absence of impact decreased to 25% in men aged 35–44 years from 63% in men aged 18–24 years. Likewise, absence of impact on sexual life decreased with HPV awareness from 43.4% to 26.4% (*p* value 0.05) (Table 4).

**Table 4:** Patients impact on sexual life associated with genital warts, men

	**Concern and impact on sexual life (%)**					**Chi square**
	**n**	**Absent**	**Mild**	**Moderate**	**Severe**	***p *value**
**Age**						
18-24	19	63.2	0.0	10.5	26.3	
25-34	63	30.2	1.6	14.3	54.0	
35-44	24	25.0	12.5	20.8	41.7	0.03
**Education (high level)**						
Primary	6	33.3	0.0	16.7	50.0	
Secondary, High-school	51	25.5	5.9	11.8	56.9	
University	25	52.0	4.0	16.0	28.0	
Postgraduate	24	37.5	0.0	20.8	41.7	0.34
**Civil status**						
Single	68	33.8	5.9	14.7	45.6	
Married	26	38.5	0.0	15.4	46.2	
Divorced	**4**	50.0	0.0	25.0	50.0	
**Awareness of HPV**						
Yes	53	26.4	7.6	13.2	52.8	
No	78	38.5	5.1	12.8	43.6	0.67
**Location**						
Single Location	81	33.33	7.4	13.58	45.7	
Multiple location	25	40	8.0	4.00	48.0	0.62
Specific Location of GW*						
**Glans/sulcus**	9	22.2	0.0	11.1	66.7	
Shaft	42	26.2	7.1	16.7	50.0	
Foreskin	24	50.0	12.5	12.5	25.0	
Perineum	6	33.3	0.0	0.0	66.7	0.3
**Life- time sexual partners**	33.3	0.0	0.0	66.7		
1 to 4	8	25.0	0.0	37.5	37.5	
5 to 9	43	25.6	2.3	11.6	60.5	
10 plus	55	43.6	5.5	14.6	36.4	0.14

* Based on cases with lesions in a single anatomic location.

In contrast, among women, education and anatomic location of GW were associated with GW impact on sex life (Table 5). Impact on sexual life was significantly higher among those with high and postgraduate educational levels, as well as among those whose warts were located either in the labia or perineum, compared to those who had them in the introitus (83% vs. 66%; p 0.05) (Table 5).

**Table 5:** Patients impact on sexual life associated with genital warts, women

	**Concern and impact o sexual life (%)**					
	**n**	**Absent**	**Light**	**Moderate**	**Severe**	***p *value**
**Age**						
18-24	86	7.0	3.5	16.3	73.3	
25-34	54	7.4	7.4	3.7	81.5	
35-44	15	6.7	13.3	0.0	80.0	0.11
**Education (highest level)**						
Primary	11	18.2	9.1	0.0	72.7	
Secondary, High-school	103	2.9	2.9	12.6	81.6	
University	5	0.0	20.0	0.0	80.0	0.02
**Civil Status**						
Single	106	9.4	3.8	11.3	75.5	
Married	27	3.7	14.8	3.7	77.8	
Divorced	**5**	0.0	0.0	0.0	100.0	
Cohabitation	**16**	0.0	6.3	18.8	75.0	0.50
**Awareness of HPV**						
Yes	76	4.0	7.9	10.5	77.6	
No	79	10.1	3.8	10.1	76.0	0.37
**Location**						
Single location	130	6.15	8.46	10	75.38	
Multiple location	25	12	0.00	4.00	84.00	0.24
Specific Location of GW*						
Labia	65	0.0	6.2	10.77	83.08	
V. introitus	59	13.6	10.2	10.17	66.1	
Perineum	6	0.0	16.7	0	83.33	0.05
**Life- time sexual partners**						
1 to 4	117	6.0	6.0	10.3	77.8	
5 to 9	**34**	11.8	2.9	11.8	73.5	
10+	**4**	0.0	25.0	0.0	75.0	0.52

*Based on cases with lesions anatomic location.

## Discussion

The population studied were young adult patients confirming the higher prevalence of GW among young population [1]. Half of the patients declared that they had heard about HPV. This result is lower than awareness of HPV in general population in an international comparison (61%) [12] and contrasts with data indicating very low awareness of HPV in a previous Colombian study [9], but is similar to a nation-wide survey where 44% of the women between 13 and 69 years of age had heard about the HPV virus [13]. The data are also similar to a study in Latin American immigrants in the United States, where 47% of women surveyed were aware of HPV [21]. Nevertheless, considering that the study population consisted of patients with GW, the results indicate a low level of knowledge of HPV, as has been reported in a systematic review [8]. In Australia, 63% of women are aware of HPV while another study of Latin American women found 65% awareness of the virus [22,23].

We found no differences in HPV awareness based on gender or age. Although reports in the literature vary on whether sex and age are associated with HPV knowledge [21,23,24], the bottom line of most results on HPV awareness is poor knowledge about HPV in both men and women, independent of age [8,25]. In our study, more than 80% of the patients knew that HPV can cause cervical cancer and almost all knew that it can cause genital warts. This result show higher knowledge on HPV as a cause of cervical cancer than a report among Hispanic women in US [26] but similar results to a German study where 97% of young women knew that HPV causes cervical cancer [11]. On the contrary, in the same study only 49% of the population knew that HPV causes genital warts, which contrast with the results of our study. It has to be considered that in the present study patients already had genital warts and an information bias due to the procedures cannot be ruled out, as the interview was performed after the biopsy had taken place. Although almost all patients aware of HPV knew that HPV was sexually transmitted, only half of them actually knew that HPV infection can be transmitted through non-penetrating sexual intercourse. This highlights the importance of educating the population about HPV. In fact, education level was the only factor positively associated with HPV awareness in men and women in our study. Higher levels of HPV knowledge among patients with higher levels of education has also been reported elsewhere [9,14,27].

There were differences in knowledge of the HPV vaccine among men and women. In women, knowledge of the HPV vaccine was similar to knowledge of HPV. While half of women knew about the HPV vaccine, only 26% of men had heard about it. A possible explanation may be related to more frequent health consultations among women and to a closer involvement with cervical cancer screening [28], exposing them to receive more and better information on health issues and cervical cancer prevention. As for HPV, education level was also associated with HPV vaccine knowledge. This probably also explains partially that patients from the contributive regime (that in general had a better educational background) had better knowledge on HPV and the HPV vaccine than patients from the subsidized regime or patients without affiliation to the health care system.

The majority of participants reported that GW lesions influence their self-esteem and cause a severe impact on sexual life, especially in women. These results reflect the emotional distress caused by GW that has been found in several studies around the world [2,19,29,30]. A greater distress in women with external GW lesions is aligned with previous observations where the psychological impact of a sexually transmitted disease diagnosis (regardless of which one) seems to be greater in women than in men [31]. Nevertheless, our results showed that knowledge of HPV and age was associated with impact on sex life in men. Thus, older men who recognize genital warts as a sexually transmitted disease might be more affected in their sexual life. Recently, a study in the UK reported that male individuals with genital warts (age 25–44) had a lower quality of life than population controls of the same age group [32]. It is worth mentioning that the same study reported a higher impact among women, but without significant variation with age. Although that study observed a recovery trend in quality of life scores after clearance of genital warts, it was not significant, suggesting that the negative impact of genital warts may still be present long after clearance.

A strength of this study is the fact that the study included individuals from a broad age group with a diagnosis of GW. Most previous reports have been based on people with previous diagnoses of GW or general population samples (most focusing on students). To our knowledge it is the first study in Latin America evaluating awareness, knowledge and attitudes towards HPV in male and female patients affected by GW. The majority of the studies in our region have focused on cervical cancer and pre neoplasic lesions. The main limitation of the present study is that we didn’t use a pre-established validated questionnaire nor any cognitive testing or focus groups to assess the impact of GW in individuals. As such, ratings of the impact on sexual life remain subjective and without explanation on the issues lying behind. However, we explored 2 of the domains included in such instruments [33] that have shown significant relation to genital warts in other studies [15,32-34]. However, our results are consistent with the literature and could be a starting point for further evaluations of health quality life impact. Finally, the patients attending the out-patient clinic where the study was performed may not represent all patients with genital warts in the country.

## Conclusions

We found evidence of low HPV awareness in patients with GW. No differences in HPV knowledge were seen among different gender or age strata. Despite HPV awareness, GW affect both, self-esteem and sexual life in women and in older men, who identify GW as a sexually transmitted disease. This information is valuable to highlight the burden of HPV beyond cervical cancer in Latin American populations and encourages further research in this field. It also clarifies the benefits and needs of HPV prevention through education and vaccination.

## Endnotes

^a^Profamilia (Asociación Pro-bienestar de la Familia Colombiana) is a private organization, specialized in sexual and reproductive health that offers medical services throughout the country.

## Competing interests

Marion Piñeros has been member of an Advisory Board of MSD for HPV vaccine and has received travel grants from MSD for attending scientific meetings; Gustavo Hernández has received travel grants from MSD for attending scientific meetings; Gonzalo Pérez is employee at Merck&Co, INC.; the other authors declared no conflicts of interest. IJME formats have been uploaded for each author.

## Authors’ contributions

MP participated in the design of the study, supervision, analysis and writing of the present manuscript. GHS participated in the design of the study, conduction, supervision, data processing, analysis, critical appraisal of the manuscript and writing of some parts of the article. LO participated in the pilot study, data collection, interviewing of patients, quality review and critical appraisal of the manuscript. JCV participated in the pilot study, data collection and critical appraisal of the manuscript. GP participated in the conception of the study, design of the study, data review and critical appraisal of the manuscript. All authors read and approved the final manuscript.

## Pre-publication history

The pre-publication history for this paper can be accessed here:

http://www.biomedcentral.com/1471-2458/13/272/prepub

## References

[B1] Kjaer SK Sigurdsson K Iversen OE Hernandez-Avila M Wheeler CM Perez G A pooled analysis of continued prophylactic efficacy of quadrivalent human papillomavirus (Types 6/11/16/18) vaccine against high-grade cervical and external genital lesions Canc Prev Res (Phila) 2009 2 10 868 878 10.1158/1940-6207.CAPR-09-0031 19789295

[B2] Maw RD Reitano M Roy M An international survey of patients with genital warts: perceptions regarding treatment and impact on lifestyle Int J STD AIDS 1998 9 10 571 578 10.1258/0956462981921143 9819106

[B3] Insinga RP Dasbach EJ Elbasha EH Epidemiologic natural history and clinical management of Human Papillomavirus (HPV) Disease: a critical and systematic review of the literature in the development of an HPV dynamic transmission model BMC Infect Dis 2009 9 119 10.1186/1471-2334-9-119 19640281 PMC2728100

[B4] Koutsky L Epidemiology of genital human papillomavirus infection Am J Med 1997 102 5A 3 8 9217656 10.1016/s0002-9343(97)00177-0

[B5] Brown DR Schroeder JM Bryan JT Stoler MH Fife KH Detection of multiple human papillomavirus types in Condylomata acuminata lesions from otherwise healthy and immunosuppressed patients J Clin Microbiol 1999 37 10 3316 3322 10488198 10.1128/jcm.37.10.3316-3322.1999 PMC85555

[B6] Donovan B Franklin N Guy R Grulich AE Regan DG Ali H Quadrivalent human papillomavirus vaccination and trends in genital warts in Australia: analysis of national sentinel surveillance data Lancet Infect Dis 2010 S1473–3099 10 70225-5 21067976 10.1016/S1473-3099(10)70225-5

[B7] Ferlay J Shin HR Bray F Forman D Mathers C Parkin D GLOBOCAN 2008, Cancer Incidence and Mortality Worldwide: IARC CancerBase No. 10 [Internet] 2010 International Agency for Research on Cancer: Lyon, France http://globocan.iarc.fr

[B8] Klug SJ Hukelmann M Blettner M Knowledge about infection with human papillomavirus: a systematic review Prev Med 2008 46 2 87 98 10.1016/j.ypmed.2007.09.003 17942147

[B9] Hanisch R Gustat J Hagensee ME Baena A Salazar JE Castro MV Knowledge of Pap screening and human papillomavirus among women attending clinics in Medellin, Colombia Int J Gynecol Canc 2008 18 5 1020 1026 10.1111/j.1525-1438.2007.01131.x 18021221

[B10] Wiesner C Pineros M Trujillo LM Cortes C Ardila J [Human papillomavirus (HPV) vaccine acceptability amongst parents of adolescents in four Colombian areas] Rev Salud Publica (Bogota) 2010 12 6 961 973 22030683

[B11] Kuznetsov AV Muller RA Ruzicka T Herzinger T Kuznetsov L Knowledge of sexually transmitted HPV infection, genitoanal warts, cancer and their prevention among young females after vaccine introduction in Germany J Eur Acad Dermatol Venereol 2012 10.1111/jdv.12045 23216713

[B12] Marlow LA Zimet GD McCaffery KJ Ostini R Waller J Knowledge of human papillomavirus (HPV) and HPV vaccination: An international comparison Vaccine 2013 31 763 769 10.1016/j.vaccine.2012.11.083 23246310

[B13] Encuesta Nacional de Demografia y Salud, ENDS 2011 Bogotá, Colombia: Profamilia

[B14] Moreira ED Jr de Oliveira BG Neves RC Costa S Karic G Filho JO Assessment of knowledge and attitudes of young uninsured women toward human papillomavirus vaccination and clinical trials J Pediatr Adolesc Gynecol 2006 19 2 81 87 10.1016/j.jpag.2006.01.003 16624694

[B15] Woodhall SC Jit M Soldan K Kinghorn G Gilson R Nathan M The impact of genital warts: loss of quality of life and cost of treatment in eight sexual health clinics in the UK Sex Transm Infect 2011 87 6 458 463 10.1136/sextrans-2011-050073 21636616 PMC3253069

[B16] Waller J Marlow LA Wardle J The association between knowledge of HPV and feelings of stigma, shame and anxiety Sex Transm Infect 2007 83 2 155 159 17098767 10.1136/sti.2006.023333 PMC2598611

[B17] Jeynes C Chung MC Challenor R 'Shame on you'--the psychosocial impact of genital warts Int J STD AIDS 2009 20 8 557 560 10.1258/ijsa.2008.008412 19625588

[B18] Ireland JA Reid M Powell R Petrie KJ The role of illness perceptions: psychological distress and treatment-seeking delay in patients with genital warts Int J STD AIDS 2005 16 10 667 670 10.1258/095646205774357334 16212712

[B19] Clarke P Ebel C Catotti DN Stewart S The psychosocial impact of human papillomavirus infection: implications for health care providers Int J STD AIDS 1996 7 3 197 200 10.1258/0956462961917618 8799782

[B20] Mortensen GL Larsen HK The quality of life of patients with genital warts: a qualitative study BMC Publ Health 2010 10 113 10.1186/1471-2458-10-113 PMC2848198 20205944

[B21] Drewry J Garces-Palacio IC Scarinci I Awareness and knowledge about human papillomavirus among Latina immigrants Ethn Dis 2010 20 4 327 333 21305817 PMC3097024

[B22] Kobetz E Kornfeld J Vanderpool RC Finney Rutten LJ Parekh N O'Bryan G Knowledge of HPV among United States Hispanic women: opportunities and challenges for cancer prevention J Health Commun 2010 15 Suppl 3 22 29 21154081 10.1080/10810730.2010.522695 PMC3858859

[B23] Pitts MK Heywood W Ryall R Smith AM Shelley JM Richters J Knowledge of human papillomavirus (HPV) and the HPV vaccine in a national sample of Australian men and women Sex Health 2010 7 3 299 303 10.1071/SH09150 20719218

[B24] Colon-Lopez V Ortiz AP Palefsky J Burden of human papillomavirus infection and related comorbidities in men: implications for research, disease prevention and health promotion among Hispanic men P R Health Sci J 2010 29 3 232 240 20799510 PMC3038604

[B25] Walsh CD Gera A Shah M Sharma A Powell JE Wilson S Public knowledge and attitudes towards Human Papilloma Virus (HPV) vaccination BMC Publ Health 2008 8 368 10.1186/1471-2458-8-368 PMC2579427 18947430

[B26] Tiro JA Meissner HI Kobrin S Chollette V What do women in the U.S. know about human papillomavirus and cervical cancer? Cancer Epidemiol Biomarkers Prev 2007 16 288 294 10.1158/1055-9965.EPI-06-0756 17267388

[B27] Benning BR Lund MR Patient knowledge about human papillomavirus and relationship to history of abnormal Papanicolaou test results J Low Genit Tract Dis 2007 11 1 29 34 17194948 10.1097/01.lgt.0000236969.27502.71

[B28] Sach TH Whynes DK Men and women: beliefs about cancer and about screening BMC Publ Health 2009 9 431 10.1186/1471-2458-9-431 PMC2789733 19930703

[B29] Persson G Dahlof LG Krantz I Physical and psychological effects of anogenital warts on female patients Sex Transm Dis 1993 20 1 10 13 10.1097/00007435-199301000-00003 8430353

[B30] Dediol I Buljan M Vurnek AIM Bulat V A Itum M A Ubrilovia A Psychological burden of anogenital warts J Eur Acad Dermatol Venereol 2009 23 9 1035 1038 10.1111/j.1468-3083.2009.03242.x 19470067

[B31] Ikkos G Fitzpatrick R Frost D Nazeer S Psychological disturbance and illness behaviour in a clinic for sexually transmitted diseases Br J Med Psychol 1987 60 Pt 2 121 126 3620388 10.1111/j.2044-8341.1987.tb02721.x

[B32] Drolet M Brisson M Maunsell E Franco EL Coutlee F Ferenczy A The impact of anogenital warts on health-related quality of life: a 6-month prospective study Sex Transm Dis 2011 38 10 949 956 10.1097/OLQ.0b013e3182215512 21934571

[B33] Mast TC Zhu X Demuro-Mercon C Cummings HW Sings HL Ferris DG Development and psychometric properties of the HPV Impact Profile (HIP) to assess the psychosocial burden of HPV Curr Med Res Opin 2009 25 11 2609 2619 19739938 10.1185/03007990903238786

[B34] Pirotta M Ung L Stein A Conway EL Mast TC Fairley CK The psychosocial burden of human papillomavirus related disease and screening interventions Sex Transm Infect 2009 85 7 508 513 10.1136/sti.2009.037028 19703844

